# Comments on: A functional polymorphism rs10830963 in melatonin receptor 1B associated with the risk of gestational diabetes mellitus

**DOI:** 10.1042/BSR20194316

**Published:** 2020-02-20

**Authors:** Klara Rosta, Jürgen Harreiter, Ákos Nádasdi, László Németh, Alexandra Kautzky-Willer, Anikó Somogyi, Gábor Firneisz

**Affiliations:** 1 Department of Obstetrics and Gynecology, Medical University of Vienna, Austria; 2 1^st^ Department of Obstetrics and Gynecology, Semmelweis University, Budapest, Hungary; 3 Gender Medicine Unit, Division of Endocrinology and Metabolism, Department of Internal Medicine III, Medical University of Vienna, Austria; 4 2^nd^ Department of Internal Medicine, Semmelweis University, Budapest, Hungary; 5 Department of Probability Theory and Statistics, Eötvös Loránd University, Budapest, Hungary; 6 MTA-SE Molecular Medicine Research Group, Hungarian Academy of Sciences - Semmelweis University, Budapest, Hungary

**Keywords:** GDM, Gene variant, Gestational diabetes mellitus, meta analysis, MTNR1B

## Abstract

We have read with great interest the accepted manuscript of the meta-analysis performed by Huang, et al. titled “A functional polymorphism rs10830963 in *melatonin receptor 1B* associated with the risk of gestational diabetes mellitus” published online in the 2019 December 6 issue of *Bioscience Reports* (https://doi.org/10.1042/BSR20190744).

We do agree with the authors’ final conclusion that such a meta-analysis should eventually confirm that the *MTNR1B* rs10830963 *G* allele is significantly associated with increased risk of gestational diabetes mellitus (GDM) development in pregnant populations with Asian and European ancestry. However we have surprisingly found that our genetic association study (*PLoS One* (2017), https://doi.org/10.1371/journal.pone.0169781) was included in this meta-analysis, but with mistakenly calculated odds ratios (OR). Therefore we would suggest to use the correct OR values based on our original publication that were already indicating a high genetic effect size for the *MTNR1B* rs10830963 risk variant on GDM development.

Dear Editor,

We have published the OR = 1.84 (95% CI:1.54±2.21), p = 7x10^−4^ value to characterize the association of the *MTNR1B* rs10830963 *G* allele and GDM diagnosis using the IADPSG criteria under the dominant genetic model and in addition the OR and p values were also reported for the genetic association using the modified 1999’ WHO GDM diagnostic criteria and also under the additive model [2].

Conflictingly, the authors reported “0.99-0.99-0.99” as OR values [1] - of unknown origin - for our study (labelled as “Rosta et al.” on Figures 2-3-4) in the forest plots on the risk of GDM associated with rs10830963 (“*CG* vs. *CC”* and “*GG vs CC*” and “*C* vs *G*”, respectively). Despite that we did specifically report the minor (*G*) allele frequencies (MAF) in our study as 0.36 in the GDM group and 0.28 in the control group, these MAF data were apparently also wrongly indicated in the meta-analysis (MAF values of “0.33 and 0.30” in Table 1, respectively).

The correct (originally published) MAF values could have been directly used to calculate the allelic (rs10830963 *G*) OR value. Our published data were sufficient to make accurate further OR estimations and calculating the number of individuals with the *MTNR1B* rs10830963 *CC, CG* and *GG* genotypes in both the GDM and control study groups. We directly reported 8 distinct OR values for all the 77 gene variants - including the *MTNR1B* rs10830963 - assessed in our study (under the additive and the dominant genetic models; using both the IADPSG and the m’99 WHO criteria; with adjustments to age only and to age and pre-pregnancy BMI also - see S2 table of the original publication [2]).

In order to help the meta-analysis of Huang et al. hereby we report the correct results for those comparisons that should have been used in this meta-analysis regarding our study, i.e. the valid OR and crude p for the *CC* homozygous vs *CG* heterozygous; for the *CC* homozygous vs *GG* heterozygous groups and the allelic OR values are as follows (95% CIs were calculated using the 10000 bootsrap simulations):

*CC* vs *CG*: OR = 1.85 (95%CI: 1.06-2.37) p = 0.001
*CC* vs *GG*: OR = 1.81 (95%: 0.72- 3.09) p = 0.05
*C* vs *G*: OR = 1.44 (95%CI: 0.79 - 1.26) p = 0.026

with the case/control numbers for the *MTNR1B* rs10830963 variant under the IADPSG criteria (as published): 284/485.

There are further points (including the Table 1-3, Figures 2-3-4-5 abstract and the text) that would require an update due to that the copy (MAF) and calculation (OR) errors as described above may affect the overall result (for the Caucasian populations) of the meta-analysis.

Such an extensive update might provide the authors with a very welcome opportunity to correct the other mistakes in the article, such as the clinical table, where they inaccurately characterized our control pregnant population, despite all participants were assessed with a 75g OGTT in both countries and the controls were defined as those pregnants who did not merit the studied GDM diagnostic criteria as we have described. In addition the weighted mean pre-pregnancy BMI and age values of the Hungarian and Austrian pregnant populations could also be calculated from the original publication [2].
